# The Association between Trust in Health Care Providers and Medication Adherence among Black Women with Hypertension

**DOI:** 10.3389/fpubh.2013.00066

**Published:** 2013-12-05

**Authors:** Willie M. Abel, Jimmy T. Efird

**Affiliations:** ^1^School of Nursing, University of North Carolina at Charlotte, Charlotte, NC, USA; ^2^Department of Cardiovascular Sciences, Brody School of Medicine, East Carolina Heart Institute, East Carolina University, Greenville, NC, USA

**Keywords:** trust, medication adherence, Black women, hypertension, health care provider

## Abstract

**Background**: Black women have the highest prevalence of hypertension in the world. Reasons for this disparity are poorly understood. The historical legacy of medical maltreatment of Blacks in the U.S. provides some insight into distrust in the medical profession, refusal of treatment, and poor adherence to treatment regimens.

**Methods**: Black women (*N* = 80) who were prescribed antihypertensive medications were recruited from urban communities in North Carolina. Study participants completed the Trust in Physician and Hill-Bone Compliance to High Blood Pressure Therapy questionnaires. An exact discrete-event model was used to examine the relationship between trust and medication adherence.

**Results**: Mean age of study participants was 48 ± 9.2 years. The majority of participants (67%) were actively employed and 30% had incomes at or below the federal poverty level. Increasing levels of trust in the health care provider was independently associated with greater medication adherence (*P*_Trend_ = 0.015).

**Conclusion**: Black women with hypertension who trusted their health care providers were more likely to be adherent with their prescribed antihypertensive medications than those who did not trust their health care providers. Findings suggest that trusting relationships between Black women and health care providers are important to decreasing disparate rates of hypertension.

## Introduction

Mutual trust is essential to the patient/heath care provider relationship and the achievement of positive health outcomes. To be trustworthy, health care providers need to show competence, caring behaviors, good interpersonal skills, and a desire to promote the health of the patients they serve (1, 2). Likewise, trustworthy patients need to be honest, adhere to the treatment regimen, and perform self-care behaviors (2). Trust is not assumed, but it can be earned over time as the patient and health care provider get to know each other by working together to ensure that accurate medical conclusions are obtained and the best course of treatment is determined and executed (1). Similarly, a patient’s trust in the health care provider implies confidence that their words are truthful and actions are appropriate in the provision of care and treatment (1). When health care providers trust their patients, it implies a belief that the patient will seek timely health care, reveal sensitive information, and follow the recommended treatment regimen (1).

The historical legacy of Blacks in the U.S. provides insight as to why distrust in the medical profession exists. Introduced to the U.S. as slaves, Blacks had little to no human rights. Frequently, they were used in experiments by White doctors to perfect medical and surgical techniques before attempting procedures on Whites (3–5). Because of skin color and other distinctive features such as hair texture, thick lips, and body shape, the lives of Blacks were not valued, and their exploitation by White physicians endured a long history (3–5). Myths were invented by the medical community to portray Blacks as different, inferior, and less than human. These myths resulted in the enactment of laws in the U.S. that distinguished Black people as property on the same level with livestock and beast of burden (5, 6). Published journal articles and doctors records on various types of medical experimentation with Blacks spanned several centuries. Examples of experiments included: unanesthetized gynecological experiments on slave women; Tuskegee syphilis study that withheld treatment to plot disease progress in men; eugenic-inspired involuntary sterilizations of welfare mothers with multiple children; unconsented high dose radiation experiments; and hazardous dermatological research on prison subjects (4).

Present treatment related issues also influence distrustful relationships between Blacks and health care providers. In the landmark Institute of Medicine report (7, 8), research indicated that minority/ethnic groups are less likely than Whites to receive needed services, procedures, and routine treatments for common health problems and for diseases such as cancer, cardiovascular disease, and diabetes. In cardiac care for example, minorities are less likely to receive pharmacological therapy, transplants, catheterization, and bypass surgery even when treatments and procedures are judged to be appropriate (7, 8). In contrast, they are more likely to receive lower quality care and undesirable procedures such as lower extremity amputations for diabetes. Equally perplexing is the strong link related to finances and access to cardiac care (9) that often results in discriminatory practices for poor people. It is also daunting to consider that discriminatory practices persist even in Blacks and other minority/ethnic groups who have the ability to pay for health care services (10). Thus, the maltreatment experienced by Blacks in the health care arena contributes not only to distrust, but also refusal of treatment and poor adherence to treatment regimens (3, 7). Today, remnants of history continue in subtle configurations resulting in many Blacks approaching health care with fear, skepticism, and caution (3).

Currently, disparate rates of heart disease, kidney disease, and stroke associated with uncontrolled hypertension (HTN) in Blacks who reside in the U.S are perplexing. When the prevalence of HTN is delineated by race and sex, HTN is greater for Black women (47%), followed by Black men (43%), then White men (33%) and White women (31%), and is lowest for Hispanic men (30%) and women (29%) (11). These statistics are especially concerning because Black women are cited as having the highest prevalence of HTN in the U.S. and worldwide, and rates are increasing (11). Thus, alleviating sources that potentiate non-adherence to treatment regimens such as medication adherence is important. One factor, mistrust in health care providers among Black women with hypertension may potentially lead to poorer medication adherence. To our knowledge, no studies have examined the association of trust and adherence to antihypertensive medications in an exclusively Black female population. Therefore, this study examined the association between trust in health care providers and medication adherence among these Black women.

## Materials and Methods

### Study sample

A cross-sectional pilot study was conducted with hypertensive Black women in the Piedmont region of North Carolina. Methods used in this study have been reported elsewhere and are briefly described here (12). Inclusion criteria included (a) ages ranging from 18 to 60 years, (b) taking one or more prescription medication(s) for HTN, and (c) English speaking. Exclusion criteria included self-report of (a) mental illness that interfered with daily functioning, (b) current pregnancy, or (c) concurrent participation in another research study. Using an *a priori* adaptive algorithm to compute power, a sample size of 80 was required to detect an effect size of 4.5 (alpha of 0.05) with power of 80.9%, assuming a referent event rate of 0.4 (13). Recruitment efforts included distributing flyers to various Black communities and businesses such as hair salons, churches, and community events, along with social nomination. Informed consent was obtained from all participants who met eligibility criteria and agreed to participate in the study. Data were collected in a private setting to ensure participant privacy. Questionnaires were generally completed in a 1-hour session. Approval was obtained from the Institutional review board at the University of North Carolina at Greensboro.

### Measures

Standardized instruments were used to gather information on medication adherence and trust in the health care provider among study participants. Demographic data (e.g., education, income, medical history, etc.) and anthropometric measurements (e.g., blood pressure, height, weight, and waist circumference) also were collected.

#### Trust in Physician Scale

The Trust in Physician Scale (TPS) is an 11-item tool that measures patient trust in their physician (14). In the current study, the word “physician” was replaced with “health care provider” because of the likelihood that patients would be seen by mid-level providers such as nurse practitioners or physician assistants. Responses on the TPS are scored on a five-point Likert scale from 1 (strongly agree) to 5 (strongly disagree). “Neutral” was not used as a response item. This was done in an effort to force participants to express their opinion to specific responses because neutral data would not provide meaningful information. Scores can range from 11 to 55 with higher scores indicating greater trust. Four items are reverse scored to prevent repetitive responses. Cronbach’s alphas for the TPS ranged from 0.85 to 0.90 in a previous study (14) and alpha was 0.835 for this study.

#### Hill-Bone Compliance to High Blood Pressure Therapy Scale

The Hill-Bone Compliance to High Blood Pressure Therapy Scale (CHBPTS) is a 14-item tool used to assess antihypertensive medication adherence and has been reported elsewhere (12). In this study, “compliance” was replaced by “adherence” because of the passivity associated with the word “compliance” that hinders the establishment of a working relationship with the person (15, 16). Included in the Hill-Bone CHBPTS is the four-item Medication Adherence Scale (MMAS) developed by Morisky et al. (17, 18). The Hill-Bone CHBPTS includes three subscales, and for this study only the medication subscale (eight items) was chosen to assess medication-taking behavior plus one item that addressed prescription refills. Responses are scored on a four-point Likert scale from 1 (“none of the time”) to 4 (“all the time”). The minimal medication-taking score is 9 and the maximum score is 36. Lower scores reflect medication adherence behaviors and higher scores represent non-adherence. Cronbach’s alpha for the medication subscale was 0.77 in a previous study (19) and alpha was 0.843 for this study.

### Data analysis

Descriptive statistics were used to characterize the sample and study tools. Cronbach’s alpha measured internal reliability of the instruments and a consistency of 0.70 or higher are considered acceptable and values of 0.60 or higher are marginally acceptable (20). The exact discrete-event (proportional odds) model is a log-linear procedure that is especially suited for analyzing ordinal outcomes with non-normally distributed error terms (21). Relative importance scores (RIS), denoting the proportional odds of a higher (or lower) outcome score for a specific level of a predictor variable compared with referent group, were computed using this model. *P* values for linear trend across levels of a predictor variable were computed using a likelihood ratio test. Variables were included in the optimal predictive model if, for at least one level of a predictor variable, *p* < 0.025 and RIS >2.5 or RIS <0.4. The *post hoc* addition of other variables into the final model was determined in a systematic, pairwise fashion, based upon a 10% increase or decrease in the magnitude of levels for the main effect variable (e.g., trust in the health care provider). All statistics were computed using SAS version 9.3 (Cary, NC, USA).

## Results

Participants in the sample ranged in age from 19 to 60 years, with the majority aged 50 or older. Approximately 68% were not married, 33% were unemployed or retired, and over 70% were obese. A total of 84% had completed high school or a higher level of education (see Table 1). The duration of antihypertensive medication treatment for participants ranged from 1 month to 37 years (mean 10.2 ± SD 8.6, median 10). Of the 20 participants who claimed complete adherence to their antihypertensive medications, the majority were in the 50–60 age group (*n* = 12). The instruments used in the study had acceptable internal reliability (Cronbach’s alpha ≥60%). The median score for participant’s trust in their primary health care provider was above the mean of possible values (mean 43.9 ± SD 6.3, median 45.0), with a score of 55 indicating perfect trust. The median score for participant’s adherence to antihypertensive medication was below the mean of possible values (mean 13.1 ± SD 4.2, median 12.5), with a score of 9 indicating perfect adherence.

**Table 1:** **Sample characteristics and demographics (*N* = 80)**.

Characteristics	*N* (%) or mean (±SD)
Age	47.8 (±9.2)
Marital status
Single (never married)	22 (28)
Married	26 (33)
Separated	9 (11)
Divorced	16 (20)
Widowed	7 (9)
Employment status
Employed	54 (68)
Unemployed/disabled	24 (30)
Retired	2 (3)
Comorbidities	2.8 (±1.6)
Number of medications	5.1 (±3.6)
BP
SBP mean	135.2 (±19.8)
DBP mean	81.3 (±12.6)
BMI	36.8 (±10.4)
Subscales
Underweight (<18.5 kg/m^2^)	1 (1)
Normal weight (18.5–24.9 kg/m^2^)	9 (11)
Overweight (25–29.9 kg/m^2^)	10 (13)
Obesity (≥30 kg/m^2^)	60 (75)
Education
Less than 12th grade	13 (16)
High school graduate	16 (20)
GED or equivalent	1 (1)
Some community college	11 (14)
Graduated community college	6 (8)
Some 4-year college	5 (6)
Graduated 4-year college	10 (13)
Some graduate school	2 (3)
Completed graduate school	6 (8)
Completed trade or vocational school	10 (13)
Income
<$10,000	15 (19)
$10,000–14,999	4 (5)
$15,000–19,999	10 (13)
$20,000–24,999	8 (10)
$25,000–$34,999	10 (13)
$35,000–$44,999	12 (15)
$45,000–54,999	6 (8)
$55,000–64,999	6 (8)
$65,000–74,999	3 (4)
$75,000–$99,999	3 (4)
$100,000 and over	2 (3)
Refused	1 (1)

*Percentages rounded*.

*BP = blood pressure; SBP = systolic blood pressure; DBP = diastolic blood pressure; BMI = body mass index*.

Three univariable variables that predicted medication adherence were participant age, number of medications, and trust in health care provider (see Table 2). All age groups (Q2–Q4) were less likely to be adherent but participants in the 40–49 age group (Q3) had the highest RIS score and were 3.6 fold less likely to be adherent to antihypertensive medications than baseline (Q1) (*P* for trend = 0.028). In contrast participants who reported taking five to seven medications (Q3) were 3.8 fold more likely to be adherent than baseline (Q1) (*p* = 0.001). However, a significant linear trend was not observed for “number of medications.” The third univariable variable that predicted medication adherence was trust in health care provider. Participants who scored in the highest trust group Q4 (>52) were 16.7 fold more likely to be adherent than baseline (Q1) (*P* for trend = 0.010).

**Table 2:** **Exact discrete-event model for compliance scale (range = 9–33)**.^¶^

Characteristics	Univariable predictors		Optimal predictive model	
	Crude RIS	95% CI	Adjusted RIS	95% CI
Trust in primary health care provider				
Q1 (<41) (Low)	1.0	Referent	1.0	Referent
Q2 (41–45)	0.86	0.44–1.7 (*p* = 0.66)	0.84	0.40–1.8 (*p* = 0.65)
Q3 (46–52)	0.57	0.28–1.2 (*p* = 0.12)	0.64	0.29–1.4 (*p* = 0.26)
Q4 (>52) (high)	0.06	0.01–0.39 (*p* = 0.003)	0.03	0.004–0.23 (*p* = 0.0006)
		*P*_Trend_ = 0.010^§^		*P*_Trend_ = 0.015^§^
Patient age (years)
Q1 (>55)	1.0	Referent	1.0	Referent
Q2 (50–55)	2.3	1.1–4.9 (*p* = 0.029)	2.9	1.3–6.5 (*p* = 0.011)
Q3 (40–49)	3.6	1.6–8.0 (*p* = 0.001)	3.7	1.6–8.6 (*p* = 0.0028)
Q4 (<40)	2.4	1.02–5.4 (*p* = 0.044)	3.2	1.3–8.2 (*p* = 0.013)
		*P*_Trend_ = 0.028^§^		*P*_Trend_ = 0.0036^§^
No. of medications
Q1 (1–2)	1.0	Referent	1.0	Referent
Q2 (3–4)	0.56	0.28–1.1 (*p* = 0.11)	0.47	0.22–0.99 (*p* = 0.047)
Q3 (5–7)	0.26	0.12–0.59 (*p* = 0.001)	0.28	0.12–0.68 (*p* = 0.0048)
Q4 (8–18)	0.65	0.31–1.4 (*p* = 0.25)	0.73	0.34–1.6 (*p* = 0.45)
		*P*_Trend_ = 0.11^§^		*P*_Trend_ = 0.35^§^

*^¶^Lower scores reflect medication adherent behaviors and higher scores reflect non-adherent behaviors. ^§^Likelihood ratio trend test. RIS = Relative Importance Score; CI = confidence intervals; Q = quartile*.

Variables retained in the “optimal predictive model” included participant age, number of medications, and trust in health care provider (see Table 2). Again all age groups were less likely to be adherent, but participants in the 40–49 age group (Q3) had the highest RIS score and were 3.7 fold less likely to be adherent to antihypertensive medications than baseline (Q1) (*P* for trend = 0.0036). Whereas participants who reported taking five to seven medications (Q3) were 3.6 fold more likely to be adherent than baseline (Q1) (*p* = 0.0048). However, similar to the univariable case, a significant linear trend was not observed for “number of medications.” Participants who scored in the highest trust group Q4 (>52) were 33.3 fold more likely to be adherent than baseline (Q1) (*P* for trend = 0.015).

Thus, increasing levels of trust in the health care provider was associated with greater medication adherence (optimal predictive model; Q1: RIS = 1.0; Q2: RIS = 0.84, 95% CI = 0.40–1.8; Q3: RIS = 0.64, 95% CI = 0.29–1.4; Q4: RIS = 0.03, 95% CI = 0.004–0.23; *P*_Trend_ = 0.015), independent of age and number of medications (see Table 2). In summary, participant age was the variable that independently predicted non-adherence to antihypertensive medications and trust was the only variable that independently predicted adherence to antihypertensive medications in our “optimal predictive model.”

## Discussion

To date, studies on trust and medication adherence have been conducted primarily with Black men (22). Other studies have included both Black and White men and women but did not specifically target Black females (23). The current study represents the first to examine the association between trust and medication adherence in an exclusively Black female population.

Several studies have cited distrust of the medical community as the rationale for non-adherent health behaviors (2, 24–26). Blacks often mention the Tuskegee experiments and medical injustices experienced personally or by family members and friends. These injustices, along with issues such as racism, discrimination, access to care, financial barriers, thoughts of being experimented on, and substandard health care affect the ability of Blacks to trust the medical community (2). Consequently, many Blacks are reluctant to seek health care or follow medical advice resulting in non-adherence to the treatment regimen and subsequent health problems (2, 26–28). Thus, antihypertensive medication non-adherence, is likely to result in poor blood pressure control with greater organ damage such as heart failure, kidney disease, myocardial infarction, and stroke (11).

In the current study, all age groups were less likely to be adherent to their antihypertensive medications, but the greatest predictor was participants aged 40–49. These findings differ from those of other studies. For example, Schoenthaler et al. (29) reported that younger Blacks exhibited more non-adherent medication behaviors. In addition, Weingarten and Cannon (30) reported lower adherence to antihypertensive medications among those younger than age 55 and older than age 65. A higher percentage of participants over age 50 in our study indicated medication adherence compared to those less that age 50. One explanation for less adherence to antihypertensive medications among those aged 40–49 could be the stressors of marriage, working, and family responsibilities because women at this age are more likely to be married or separated, work full- or part-time, and live in households with more people than those in the other age groups.

Furthermore, participants in our study who took five to seven medications were more likely to be adherent to their medication regimen. This finding was not consistent with the literature which reports that fewer medications equate to better adherence (31, 32). Perhaps those with a higher number of medications perceived themselves as sicker and were therefore more adherent. Also, the increased number of medications may have serendipitously contributed to frequent visits to the health care provider that fostered better communication, trust, and engagement in self-care and medication adherence.

Participants in this study who reported the most trust in their health care providers were more adherent to their prescribed antihypertensive medications. This finding is consistent with the view that trust promotes better medication adherence (29, 33). Nguyen et al. (34) used the same instruments to measure trust and medication adherence as those used in the current study and found that higher adherence was associated with greater trust in the health care provider in participants with inflammatory bowel disease (IBO). One major difference between the two diagnostic groups that may influence adherence is that IBO has symptoms and HTN is silent.

The issue of poor adherence in Black women with HTN is multifactorial and complicated by cultural, psychosocial, socioeconomic, environmental, physiological, and genetic influences (35). Health care providers need to understand how these processes influence the health and behaviors of patients and potentially simulate rebellion, conflict, and mistrust in relationships with providers (36). The enormity of the multifaceted and ubiquitous nature of adherence sheds light on the limited success of adherence research and interventions. Therefore it is vital that health care providers devise strategies to alter the processes and systems that influence the health status of individuals and populations and empower groups and individuals to work on their own behalf (36). Thus, future research aimed at involving the patient as a participant in their health care may be a key factor to devising strategies that influence the multifaceted nature of non-adherence and mistrust of health care providers.

Interestingly, it is well documented that antihypertensive medications have proven efficacy in lowering blood pressure when patients adhere to the treatment regimen (37–40). However, many Black women have not readily participated in risk reduction behaviors to manage and prevent HTN (41–43). One possible explanation for the lack of adherence to treatment regimens may be attributed to the effect that past medical injustices have on present health behaviors. Distrust in health care providers is a potential barrier to medication adherence among Black women with hypertension. Our study found that increased trust was associated with greater medication adherence. Thus, it is important that health care providers and Blacks with HTN find ways to transcend the effects of history, restore trust in the health system, and form collaborative relationships to foster optimal health care.

### Limitations

Several limitations were noted in this study. The use of self-report measures may introduce personal bias. Moreover, the small convenience sample limits the generalizability of the findings to other populations in different regions of the U.S. Future studies could increase the representativeness of the sample by recruiting participants from geographically diverse sites. In addition, other factors besides trust in the health care provider may impact medication adherence in Black women with HTN. Additional research is needed to identify other contributing factors that are associated with improving medication adherence and blood pressure control among Black women. Lastly, this was a non-randomized, cross-sectional study, and causal inferences cannot be made.

## Conclusion

In this study, Black women were more likely to be adherent to antihypertensive medications if they had a higher level of trust in their health care provider. Understanding trust issues in Black women is essential when considering their disproportionate burden of HTN. Future research should explore methods for health care providers to deliver culturally sensitive care and establish trusting interpersonal relationships with Black women.

## Conflict of Interest Statement

The authors declare that the research was conducted in the absence of any commercial or financial relationships that could be construed as a potential conflict of interest.
